# PRESIDENTIAL SYMPOSIUM: HARNESSING SOCIAL NETWORKS TO OPTIMIZE ENVIRONMENTAL CONTEXTS FOR DIVERSE AGING EXPERIENCES

**DOI:** 10.1093/geroni/igz038.2163

**Published:** 2019-11-08

**Authors:** Philip A Rozario, Emily Greenfield, Nancy Kusmaul

**Affiliations:** 1 Adelphi University, Garden City, United States; 2 Rutgers University, New Brunswick, New Jersey, United States; 3 University of Maryland, Baltimore County, Baltimore, Maryland, United States

## Abstract

Social networks provide opportunities for engagement with others and structure the receipt and provision of emotional, instrumental, informational and appraisal support. Indeed scholars in this field have documented the importance of having strong social networks in influencing older adults’ well-being and quality of life. The three papers in this symposium draw on the convoy model of social relations and ecological model to examine and better understand the micro, mezzo, macro contexts that shape and influence how older people engage with and benefit from their networks in three areas: low-income senior housing communities, urban areas specifically targeting older Latinos with dementia, and disaster preparedness in micropolitan counties in eastern Iowa. The first paper, a cross-sectional study focusing on social connections in senior housing communities, examines levels of social networks, engagement, support and loneliness and their relationship with well-being outcomes. The second paper, a community-based participatory research project, reports an intervention that seeks to train natural helpers in a predominantly Latino urban neighborhood to identify and refer older Latinos with dementia to bilingual assessment services. The third paper, synthesizing findings from interventions targeting network building at the individual and state levels as well as a community-based network analysis, presents ways to strengthen networks at the mezzo and macro levels as well as environmental contexts that enable better disaster preparedness for community-based older adults. These papers will consider practice, policy and research implications in strengthening social networks and engagement to optimize older adults’ well-being in various settings.

